# A Machine Learning Approach for Studying the Comorbidities of Complex Diagnoses

**DOI:** 10.3390/bs9120122

**Published:** 2019-11-22

**Authors:** Marina Sánchez-Rico, Jesús M. Alvarado

**Affiliations:** Department of Psychobiology & Behavioral Sciences Methods, Faculty of Psychology, Universidad Complutense de Madrid, Campus de Somosaguas S/N, 28223 Pozuelo de Alarcon, Spain; jmalvara@ucm.es

**Keywords:** comorbidities, depression, UMAP, hierarchical clustering

## Abstract

The study of diagnostic associations entails a large number of methodological problems regarding the application of machine learning algorithms, collinearity and wide variability being some of the most prominent ones. To overcome these, we propose and tested the usage of uniform manifold approximation and projection (UMAP), a very recent, popular dimensionality reduction technique. We showed its effectiveness by using it on a large Spanish clinical database of patients diagnosed with depression, to whom we applied UMAP before grouping them using a hierarchical agglomerative cluster analysis. By extensively studying its behavior and results, validating them with purely unsupervised metrics, we show that they are consistent with well-known relationships, which validates the applicability of UMAP to advance the study of comorbidities.

## 1. Introduction

Healthcare data are well known for their high complexity. Working with them pertains issues almost in every field where they are needed. Among the problems listed, which include the lack of unified databases and the combination of different data sources without almost any standardized implementation, there are certain issues that make it difficult to deal with them when working with machine learning algorithms [1]. This is a term widely used in literature and usually refers to analyses characterized by being able to learn to solve specific problems. In our case, when we refer to machine learning algorithms, we are describing a specific type of tools used for data processing and its application to the healthcare field.

Electronic health records (EHR) are one of the main sources of data in the field. They typically include multiple types of clinical data (i.e., demographics, clinical diagnoses, narrative text notes, procedures and diagnoses encoding, laboratory data) and aims to contain complete records of a patient’s medical history [2]. Given its complexity, its processing offers great benefits, but it is also prone to major limitations. When working with EHR, we must face problems related to uneven data quality, the presence of both structured and unstructured data and extreme variability problems [2]. The outlook is no better with diagnostic variables, in which we also have to address the very important and dangerous issue of collinearity. Particularly on the field of diagnostic comorbidities, solving the problem of collinearity between variables is of great importance in order to be able to use machine learning algorithms appropriately. On the field of diagnostic associations for comorbidities research, analyses based on patient aggrupation have been very common for years. Historically, the main approach to the problem was based on multivariable analyses, which included techniques such as logistic or multilinear regression analyses. Over time, however, unsupervised machine learning analyses have increasingly replaced these methods, as they replace the main limitation of the former: the bias due to the need for clinical observation [3].

Cluster analysis is one of the preferred techniques for this purpose, since it allows organizing heterogeneous data in relatively homogeneous groups that allow the identification of phenotypes in the samples [4]. Although this does not mean that it is free of limitations and biases, this method is currently being applied in many health sciences fields. In this particular concept of grouping patients according to their diagnoses, though, a large number of studies on chronic obstructive pulmonary disease (COPD) stands out [3,5,6,7,8]. With these studies, knowledge of diagnoses associated with COPD has not only improved, but it has also allowed an improvement of the statistical methodology to assist studies regarding high-dimensionality healthcare data. There are also similar studies in the field of psychiatry [9], but they are still scarce and less comprehensive.

The two main different cluster analyses methods for this purposes are hierarchical and nonhierarchical [3]. The selection of the algorithm to be applied in a given case continues to generate much debate in the community, but there are certain guidelines we can follow: for example, we do know that nonhierarchical algorithms are less versatile than their counterpart. K-means in particular, a very well known nonhierarchical clustering algorithm, has proven to be less robust and, therefore, more sensitive to the noncompliance of assumptions that are very difficult to achieve in healthcare data [10].

The main issue with healthcare data regarding cluster analysis is that it works poorly when redundant or highly correlated variables are included in the algorithm as well as when the number of variables is large [11]. With diagnostic variables, which are those that raise our concerns, we often find all of these problems: a large number of dichotomous and possible unnecessary variables and, very likely, high collinearity. The latter is especially important, since it could dominate patient assignments into clusters [5].

Furthermore, it is always a good practice to inspect the data manually after the preprocessing phase of the analysis, since it allows the discovery of possible hidden patterns in the data [12]. Unfortunately, visualizing large diagnostic combinations in every patient is a complicated matter, as two-dimensional scatterplots are just not enough to display high-dimensional datasets.

These problems can be addressed by applying dimensionality reduction techniques, which aim to preserve the main structure in the data while reducing its dimensionality to a low-dimensional projection [12]. Theses methods allow us to: (a) easily discover hidden structures in the data, enabling an easier representation of them, (b) simplify the dataset by reducing the number of variables, and (c) use the outcome of orthogonal (and, therefore, uncorrelated) vectors as an input for clustering algorithms, thus avoiding collinearity issues [5].

There are several methods for dimensionality reduction, the most popular being traditional, lineal techniques, such as principal component analysis (PCA) or multiple correspondence analysis (MCA). They both project the data such that the new coordinate system best preserves the variance in the data [13], the main difference between them beingthat the former is applied to to quantitative data while MCA is intended for categorical variables. They are both very prevalent in literature, especially on COPD comorbidity studies [5,6,7,14] and gene expression research [15].

More recent dimensionality reduction methods include t-stochastic neighbor embedding (t-SNE) [16], a nonparametric, nonlinear technique which applies another principle, aiming to find a lower-dimensional projection that best preserves the similarity with the original space [12].

However, linear dimensionality reduction such as PCA is insufficient to describe the extreme variance of healthcare diagnostic data as it does n’o account for higher order, nonlinear interaction of variables [17] that are inherent in the relationships between diagnostic associations.

t-SNE offers a great low-dimensional visualization of high-dimensional data but has significant limitations: (a) It suffers from a loss of large-scale information of intercluster relationships—which means that similar patients in the original space will be close in the low-dimensional map, while patients close in the outputted map aren’t necessarily similar in the original space [12]; (b) it is unable to represent very large datasets; and (c) it has a slow computation time.

Recently, however, McInnes et al. [18] presented uniform manifold approximation and projection (UMAP), a parametric and nonlinear dimensionality reduction technique that rivals t-SNE in terms of visualization quality and is able to create informative clusters and organize them in a meaningful way.

t-SNE has been the gold standard for dimensionality reduction for many years but suffers from several constraints that UMAP has managed to address [17,19,20] (1) t-SNE suffers from a loss of large-scale information due to its inability to preserve the global structure of the original data; (2) t-SNE is designed to perform dimensionality reduction exclusively using Gaussian distance, while UMAP allows calculation with any distance formula. This provides greater flexibility when it comes to the data that can be used in the analysis; (3) one of the great limitations of t-SNE is its efficiency. Analyses tend to be very long and increase greatly in their execution time with few increases in the size of the original dataset, UMAP has proven to be a technique with much shorter runtime, and (4) UMAP has also shown more reproducible results than those obtained by t-SNE; furthermore, (5) UMAP has proved to be a very effective tool in datasets with numerous outliers, offering better results in mapping groups than t-SNE and, in a smaller degree, than PCA [21].

The aim of this study is to apply UMAP to a healthcare dataset to study its performance in grouping patients, according to their diagnoses, using an agglomerative hierarchical cluster analysis. Through a comprehensive examination of its performance and results, we hope to prove its quality of application to this field of research.

## 2. Materials and Methods

### 2.1. Data Source

The dataset used for this study was extracted from the “minimum dataset at hospital discharge” (or CMBD in Spanish). CMBD are mandatory registers of information collected by hospitals by ministry regulations. They usually include information about demographics, administrative issues (such as date, admission and discharge departments, etc.), and wide diagnosis and procedure information.

**Figure 1:**
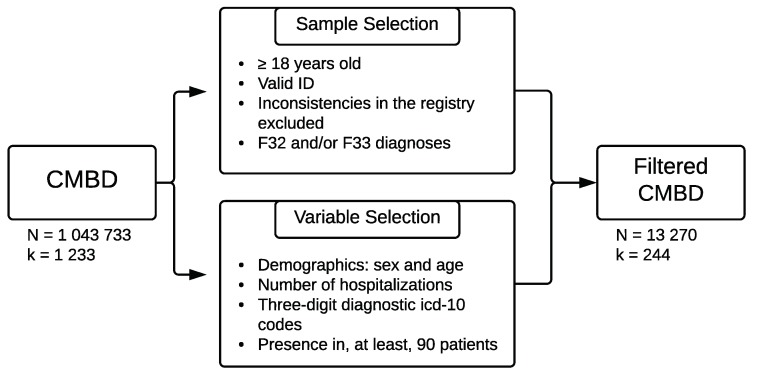
Sample and variables selection.

We extracted and preprocessed a sample of N = 13,270 depression-diagnosed patients from the aggregate CMBD databases from 2016 and 2017 [22]. Figure 1 shows the selection process for the sample and variables.

The final sample contained 241 dichotomous diagnosis variables that indicated the presence or absence of each disorder per patient. It is also relevant to indicate that the codes selected for depression were the three-digit aggrupation of F32 (Major depressive disorder, single episode) and F33 (Major depressive disorder, recurrent) [23].

### 2.2. Statistical Procedure

#### 2.2.1. Dimensionality Reduction

UMAP was tested with variations of three of its arguments: the number of dimensions, the minimum embedding distance, and the number of neighbors [18].

The number of dimensions refers to how many vectors the data will be mapped. Values between 2 and 5 dimensions were established in order to test the level of simplicity at which the resulting projection would be more stable. The minimum embedding distance indicates the probability of two similar points in the original space will end up stacked together on the algorithm outcome. Smaller values enable this to happen at the expense of losing their relationship with more distant points. In this study, we set the values to 0.1 and 0.5. Regarding the number of neighbors, we applied 15, 50, and 100. This value emphasizes the level at which the data structure is sought, where high values focus on an overall structure and smaller ones rely on a more local structure.

Every distance calculation performed on this analysis was computed using the Manhattan metric mcinnes18, choi10.

#### 2.2.2. Clustering Analysis

We implemented an agglomerative hierarchical cluster analysis with the Euclidean distance used for the dissimilarity matrix computation [24] on each of the 24 previous outcomes.

In this case, we performed the clustering by varying the clustering method (average, centroid, Ward, and complete) and the number of clusters selected between 2 and 20.

#### 2.2.3. Model Evaluation

In order to evaluate which model produced more stable clusters, we calculated the average Silhouette Coefficient (SC) index for each of the resulting 1824 previous outcomes. As a general guideline, the SC index provides a value between 0 and 1 which shows how well (or badly) each of the elements is mapped to its assigned cluster, with values closer to 1 representing a better fit [25]. With the average SC in a specific cluster, we can get a general idea of how well assigned are the elements in that group. With a mean average of every cluster SC, we obtain a mean SC that can give us a general idea of the model’s performance assigning individuals to each cluster [5].

**Figure 2:**
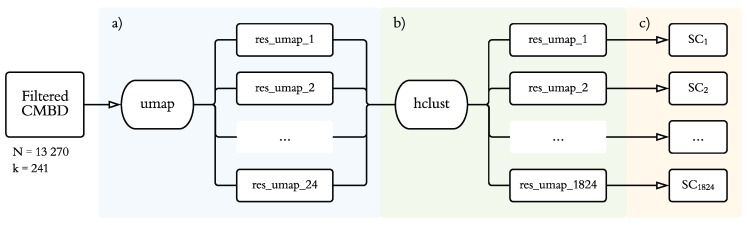
Statistical procedure. (**a**) Application of 24 combinations of uniform manifold approximation and projection (UMAP), varying the number of dimensions (2–5), minimum embedding distance (0.1, 0.5), and number of neighbors (15, 50, 100). (**b**) Application of agglomerative hierarchical clustering for each of the 1824 combinations changing the clustering method (average, centroid, Ward, and complete) and number of clusters selected (2–20). (**c**) Average silhouette coefficent for each computed model.

On Figure 2, we display the statistical analysis process carried out for this study, as explained above.

## 3. Results

### 3.1. Sample Characteristics

The sample studied consists of 13,270 patients diagnosed with clinical codes F32 (major depressive disorder, single episode) and/or F33 (major depressive disorder, recurrent) discharged from 34 hospitals in the Community of Madrid between January 2016 and December 2017. Brief demographic analyses show that the population is mostly female (with a 72.56% of women) and of advanced age (mean = 71.46, sd = 16.36, skewness = −0.77).

### 3.2. Model Selection

**Figure 3:**
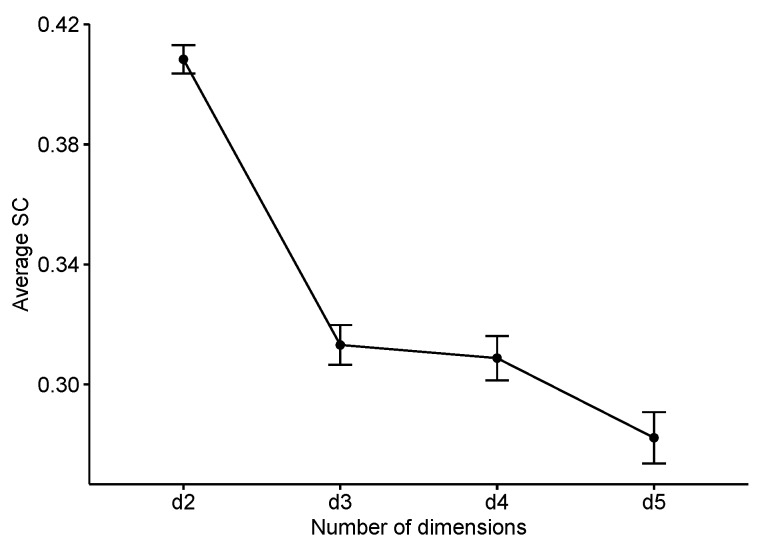
Average silhouette coefficent (SC) by number of dimensions produced by UMAP.

The first step to explore the distribution of the average silhouette coefficient was to study how it behaved depending on the number of dimensions projected by UMAP. A One-Way ANOVA (F(3, 1820) = 62.99, *p* < 0.001, $\eta_{partial}^{2}$ = 0.093) and later pairwise comparisons showed how, without a doubt, those models built from two-dimension UMAP projections were more consistent than the others (Figure 3). The same statistical analysis found that the “average” and “Ward” clustering methods also produced a higher average silhouette coefficient index (F(3, 1820) = 133.5, *p* < 0.001, $\eta_{partial}^{2}$ = 0.18).

Regarding the influence of the number of clusters influence on this index (F = 1337.7, *p* < 0.001, $\eta_{partial}^{2}$ = 0.068), models with a low number of clusters (two and three, respectively) had a much higher average silhouette value. This is, without a doubt, because the obtained two-dimensional UMAP projections—as we see in Figure 5—show three clearly different population groups. However, by choosing such a low number of clusters, we are losing a very large amount of information on the least differentiated, but also very important clusters hidden in our sample.

**Figure 4:**
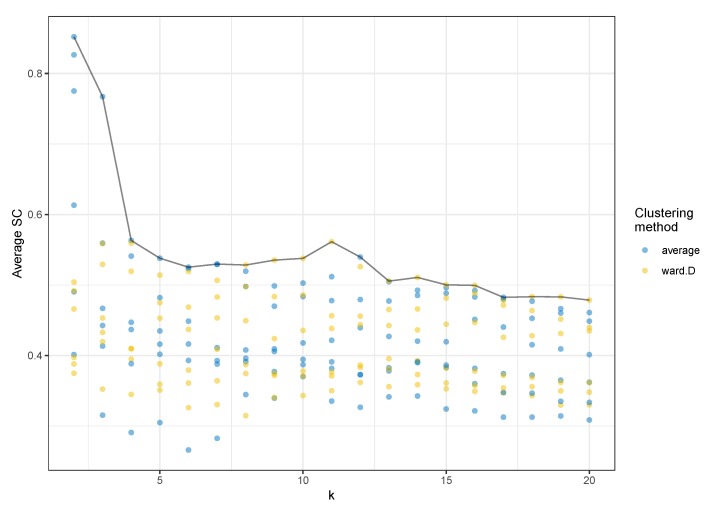
Average silhouette coefficent (SC) by number of clusters (k) and average and Ward clustering methods. For each k-value and clustering method, we can see a point for each generated model, a combination of the minimum embedding distance, and number of neighbors in the UMAP projection.

Focusing then on models with a number of clusters greater than three, we found that the one with the highest average silhouette coefficient value (SC = 0.561) was composed of 11 clusters and had been calculated with Ward’s algorithm for agglomerative hierarchical clustering (Figure 4).

**Figure 5:**
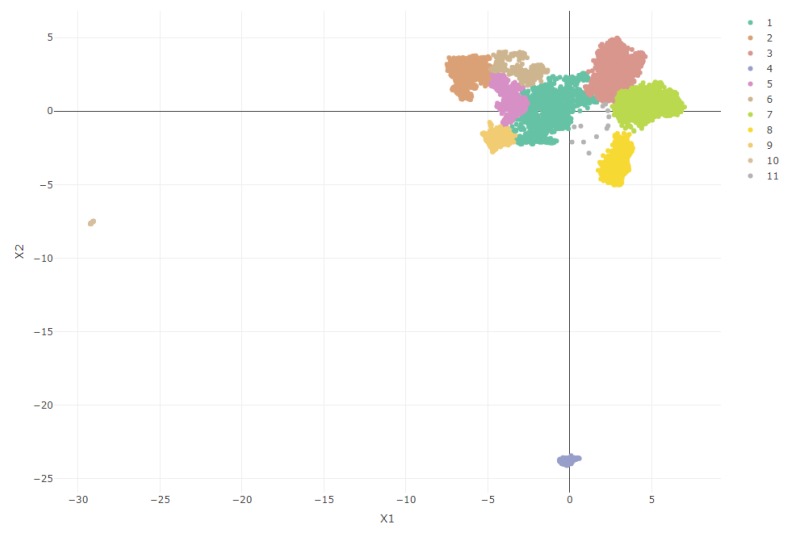
UMAP two-dimensional space projection with Ward’s clusters distribution.

Figure 5 shows a graphical representation of the sample in the two-dimensional space projected by UMAP (with 15 neighbors and a minimum embedding distance of 0.1). We also included the cluster mapping proposed by the selected model, which allows us to observe clearly the differentiated groups. Seeing these results, in which some points are far from the central core of the data, we could question whether the groups have some valid meaning to the sample or are mere outliers badly projected by the dimensionality reduction technique. However, we have two main reasons to opt for the first option: On one hand, UMAP has already shown a great capacity for outlier identification [21]. and secondly, as is discussed later in the results, these groups show a strong theoretical meaning.

### 3.3. Cluster Analysis

Once we have selected the model and observed its distribution in the low-dimensionality space, we can focus on studying the distribution and contents of the clustering outcome, aiming to check whether the model has ultimately made an accurate classification.

**Figure 6:**
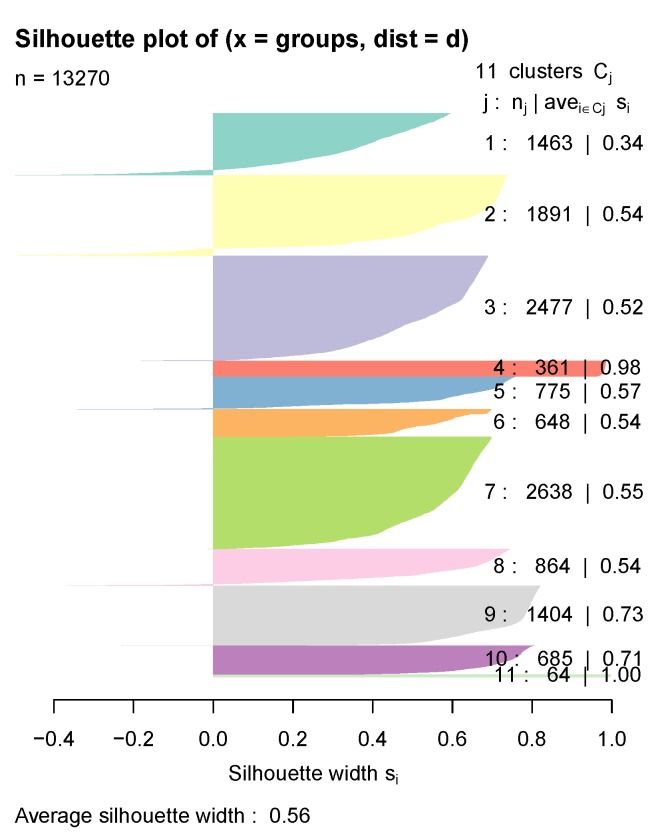
Silhouette coefficient index for each of the model selected clusters.

As can be seen in Figure 6, every cluster’s average Silhouette Coefficient is relatively high. Some negative values can be found (especially in clusters 1 and 2), suggesting patients wrongly classified, but even those clusters have a fairly decent index. Table 1 presents a summary of the model exploration, including each cluster’s main diagnosis, each group’s most relevant phenotype, and a few demographics distributions.

Cluster 1 (*Chronic diseases*, *n* = 1463) is a medium-sized cluster that has been built around a number of diagnoses previously associated with depression: endocrine diseases [26,27] and the circulatory [28,29,30,31], respiratory [6,29] and genitourinary systems [32]. Of all the clusters obtained, this is the one that includes the highest number of diagnoses with a prevalence greater than 25%, although none (with the exception of F32) is present in more than 66.2% of the patients. However, most of the most frequent associated diagnoses (E11, E78, I12, N18) have in common their chronic character, a diagnostic feature that has also been associated with depression diagnoses [33].

Cluster 2 (*No comorbidities*, *n* = 1891) is composed of patients diagnosed with a single depressive episode. Other diagnoses appear, but with a very low presence. This group therefore reflects the group of patients with a single episode of major depressive disorder without relevant association with other diagnoses.

Cluster 3 (*Primary hypertension*, *n* = 2577) is one of the largest ones and is composed primarily by the diagnosis of primary hypertension (I10), from the category of diseases of the circulatory system, also associated with depression in the literature [34].

Cluster 4 (*Major depressive disorder, recurrent*, *n* = 361), is a good example of UMAP quality, since from that projection, we can observe how it separates the population between those diagnosed with a single episode of depression (F32) and those diagnosed with recurrent major depressive disorder (F33), a classification already differentiated by the ICD-10 itself [23]. This cluster is composed of patients diagnosed with the latter diagnosis (100%). We also can appreciate endocrine disorders (33.8%) and cardiovascular disorders (31.3%) that, as we said before, are very commonly associated with depression.

Cluster 5 (*Disorders of lipoprotein metabolism and other lipidemias*, *n* = 775) shows the association between depression and endocrine and metabolic diseases described in the literature [26,27], in which the diagnosis of lipoprotein metabolism and other lipidemias (E78) appears in 96.5% together with the depressive episode, without any other associated diagnosis.

Cluster 6 (*Malignant neoplasm*, *n* = 648) refers to patients with factors influencing health status and contact with health services (Z00-Z99 codes), specifically those related to complex operations or neoplasm diagnosis.

Cluster 7 (*Metabolic disorders and hypertension*, *n* = 2638) is the largest one and associates the depressive episode with both metabolic and hypertensive disorders. As we have seen so far, these are both diagnostic groups frequently associated with depression in the literature, and this cluster reflects the group of patients presenting the three disorders.

Cluster 8 (*Cough*, *n* = 864) includes patients with cough symptomatology (98.6%). This diagnosis is a part of symptoms, signs, and abnormal clinical and laboratory findings (R00-R99). It is a very prevalent diagnosis in the population and, in theory, should not be associated with depression.

Cluster 9 (*Allergies*, *n* = 1404) presents symptoms of cough (100%), diagnostic comorbidities of primary hypertension (49.1%) and metabolic disorders (39.1%), and risk factors related to drug allergy (98.6%) [35].

Cluster 10 (*Substance addiction*, *n* = 685) includes patients with substance dependence disorders: alcohol disorders (35%) and nicotine dependence (99.3%), both previously studied as diagnoses associated with depression [29,36].

Cluster 11 (*Postpartum complications*, *n* = 64) is the smallest one and also includes a very differentiated sample of subjects in the population from the UMAP projection. It has been constructed exclusively around the ICD-10 code O99 (*Other maternal diseases classifiable elsewhere but complicating pregnancy, childbirth and the puerperium*, 100%) and includes, as can be deduced, exclusively women of a relatively early age (mean = 34.7). This sample does not include patients with postpartum depression (O90.6); therefore, what we are seeing is probably soon-to-be mothers with a single depressive episode due to pregnancy-related problems [37,38,39].

## 4. Discussion

Healthcare data are associated with collinearity problems. In this work, we aimed to address this problem through a procedure used in other fields—the application of a dimensionality reduction technique prior to a cluster analysis [5,6,7]—by applying a novel technique, UMAP [18], to a data set of Spanish adults diagnosed with depression. An agglomerative hierarchical cluster analysis was then carried out on the UMAP projections, obtaining 1824 different models, from which one was selected according to its silhouette coefficent index. The selected model was built on a two-dimensional projection and was clustered using the Ward clustering method. It identified 11 clusters that reflect diagnoses associated with Major Depressive Disorder. A detailed study of the outcomes shows the value of this tecnhique, since it reflects associations well known by previous studies in depression comorbidities.

Overall, cluster exploration shows diagnostic patterns associated with depression very similar to those already known by the theory. Especially clusters 4 (*Major depressive disorder, recurrent*) and 11 (*Pregnancy-related complications*) do a very good job of differentiating the populations included in the study. Clusters 3 (*Primary hypertension*), 5 (*Disorders of lipoprotein metabolism and other lipidemias*), and 7 (*Metabolic disorders and hypertension*) also support the quality of the model by reflecting the large association of metabolic and circulatory system disorders with depression, already known from the aforementioned studies.

The clearness of some of these results has probably been enhanced by the distinct differentiation of three different populations in the sample: patients diagnosed with recurrent major depressive disorder, patients with depressive episodes related to problems in pregnancy, and the large group of patients with a single episode of major depressive disorder. This is clearly shown in the UMAP projection and is later reflected in the distribution of the clusters and their quality.

The results of the UMAP projection are also interesting in their own right. As we previously saw, when studying the performance of the 1824 models obtained, we found that there was a big difference in the performance of those two-dimensional projections versus the rest. Moreover, we did not find any projected outcome with more than two dimensions whose clusters obtained larger silhouette values than the former. This made sense when thinking about it being the model that best fits the data. However, such a clear dichotomy in which the error variance is not enough to disrupt its order is still surprising. These results are probably due to a combination of the efficiency of the technique and the characteristics of the sample.

One of the great strengths of this study is the wide, comprehensive data set used. However, for this type of study, this data set presents one crucial limitation: This sample collects information regarding what occurred in hospital admissions during a two-year time window. This means that all previous (and, of course, posterior) diagnoses, associated or not with the disorder we wish to study, will not be reflected in the study, leading to a potential loss of information.

As for the limitations in the clustering results, the most noteworthy one is the lack of clinical experts who could make an adequate assessment of the implication of diagnoses apparently not associated with depression within these groups (i.e., R05—Cough) and of specific details of the distribution of the most populated clusters.

Regarding the model selection, this was exclusively conducted following the silhouette coefficient criteria. This is not a limitation per se, but we do consider that it would be better to add more indexes to study the models’ behavior. This is one of our current lines of research: to study the best way to assess the quality of clustering models.

Given the novelty of the UMAP, another future line of work for us would be to study the differences in quality and performance that different dimensionality reduction techniques offer in the field of diagnostic association studies.

## 5. Conclusions

The aim of this research was to examine whether UMAP is a suitable technique to apply to a healthcare dataset in order to study its performance in comorbidities association studies. For this purpose, we applied this algorithm prior to a hierarchical cluster analysis for the study of diagnostic associations. The sample contained 13,270 patients diagnosed with depression and gathered all other diagnoses for a two-year time window.

In this work, we validated the performance of UMAP with diagnostic data. We did so by studying the outcome clusters and comparing them to the results expected by the theory, finding them very similar to each other.

The main limitations of this study are the lack of experts for the evaluation of the clinical validation criterion, the restrictions of the data collection, and the inclusion of only one index criterion for the model evaluation and selection.

In spite of these limitations, we believe that this study shows the possibility of including UMAP in the field of comorbidity association, since it shows promising results on a sample of a highly comorbid diagnosis such as depression. This will benefit the research field due to the advantages of this technique over others previously known once [19].

## Figures and Tables

**Table 1:** Percentage of diagnoses coded in ICD-10 with 25% or more appearance in each cluster. The first column (Chapter name) indicates the icd10 chapter in which the corresponding diagnosis is included.

Chapter Name	3D Code	3D Name	Cl 1	Cl 2	Cl 3	Cl 4	Cl 5	Cl 6	Cl 7	Cl 8	Cl 9	Cl 10	Cl 11
Endocrine, Nutritional													
and Metabolic Diseases	E03	Other hypothyroidism						31.3					
	E11	Type 2 diabetes mellitus	39.2						39.7				
	E78	Disorders of lipoprotein											
		metabolism and lipidemias	42.0			33.8	**96.5**		**95.5**		39.1		
	E87	Other disorders of fluid,											
		electrolyte and acid-base								30.0			
Mental, Behavioral and													
Neurodevelopmental disorders	F10	Alcohol related disorders										35.5	
	F17	Nicotine dependence										**99.3**	
	F32	Major depressive disorder,											
		single episode	**99.5**	**99.9**	**99.4**		**100**	**99.8**	**99.6**	**99.9**	**99.0**	**99.6**	**100**
	F33	Major depressive disorder,											
		recurrent				**100**							
Diseases of the													
Circulatory System	I10	Primary hypertension			**99.2**	31.3			**98.0**		49.1		
	I12	Hypertensive chronic											
		kidney disease	55.5										
	I48	Atrial fibrillation flutter	37.9										
	I50	Heart faliure	43.5										
Diseases of the													
Respiratory System	J96	Respiratory failure,											
		not elsewhere classified	46.1										
Diseases of the													
Genitourinary System	N17	Acute kidney failure	39.8										
	N18	Chronic Kidney Disease	66.2										
Pregnancy, Childbirth and													
the Puerperium	O99	Other maternal diseases											
		classifiable elsewhere (...)											**100**
Symptoms, signs and abnormal													
clinical and laboratory findings	R05	Cough	55.4		32.1				38.4	**99.9**	**100**		
Factors influencing health status													
and contact with health services	Z85	Personal history of											
		malignant neoplasm						58.3					
	Z88	Allergy status to drugs,											
		medicaments (...)									**98.6**		
	Z90	Acquired absence of organs,											
		not elsewhere classified						32.3					
	Z92	Personal history of medical											
		treatment						28.7					
	Z99	Dependence on enabling											
		machines and devices (...)	31.0										

## Abbreviations

The following abbreviations are used in this manuscript:

EHR	Electronic health record
CMBD	Minimum and basic data at hospital discharge (Conjunto Mínimo Básico de Datos al alta hospitalaria)
UMAP	Uniform manifold approximation and projection
MCA	Multiple correspondence analysis
PCA	Principal component analysis
t-SNE	t-Stochastic neighbor embedding
SC	Silhouette coefficient
COPD	Chronic obstructive pulmonary disease
